# Clinical Note Creation, Binning, and Artificial Intelligence

**DOI:** 10.2196/medinform.7627

**Published:** 2017-08-03

**Authors:** Rodrigo Octávio Deliberato, Leo Anthony Celi, David J Stone

**Affiliations:** ^1^ Big Data Analytics Department Hospital Israelita Albert Einstein São Paulo Brazil; ^2^ Harvard – MIT, Division of Health Science and Technology Institute for Medical Engineering and Science Massachusetts Institute of Technology Cambridge, MA United States; ^3^ Beth Israel Deaconess Medical Center Boston, MA United States; ^4^ Departments of Anesthesiology and Neurosurgery University of Virgina School of Medicine Charlottesville, VA United States

**Keywords:** electronic health records, artificial Intelligence, clinical informatics

## Abstract

The creation of medical notes in software applications poses an intrinsic problem in workflow as the technology inherently intervenes in the processes of collecting and assembling information, as well as the production of a data-driven note that meets both individual and healthcare system requirements. In addition, the note writing applications in currently available electronic health records (EHRs) do not function to support decision making to any substantial degree. We suggest that artificial intelligence (AI) could be utilized to facilitate the workflows of the data collection and assembly processes, as well as to support the development of personalized, yet data-driven assessments and plans.

## Introduction

Many doctors find the creation of the same note more onerous in an electronic health record (EHR) than on paper [1]. The following quote from a senior physician reflects the dissatisfaction doctors have with EHRs: *“* My experience with the EHR is that it is the biggest waste of time, interferes with patient care, forces the physician to collect thousands of pieces of useless information, and produces marginal improvements in quality *”.* For this and many other reasons, the quality of EHR documentation has ranged from suboptimal to dismal [2,3]. This paper explores and envisions how artificial intelligence (AI), which is increasingly transforming facets of daily living, could support the currently burdensome process of gathering and organizing the elements necessary for the creation of a clinical note.

### Finding the Right Pieces

**Figure 1:**
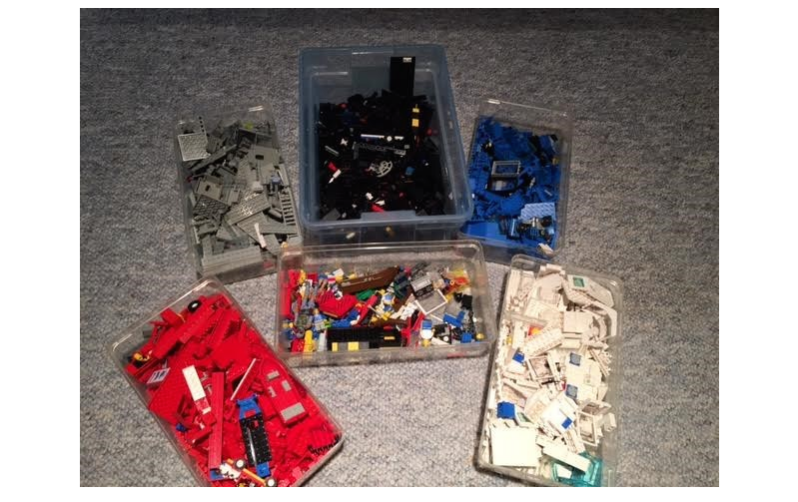
An assorted bin and 5 (mostly) color-coded bins of Lego toy pieces. The color-coded Legos may represent items that clearly and cleanly fall into a particular section of the note, depending on how the note is organized (ie, SOAP versus systems-oriented).

Part of the issue involves the user interface, where many users are not terribly facile with the keyboard and typing. It would probably be worthwhile for the creators of EHRs to design their user interfaces to be as similar as possible to the Internet-based applications, such as Web browsers, that even those who are unsophisticated with computers use every day. But the fundamental reason for this discomfort is that electronic note writers are not able to pull information seamlessly and freely from their own minds to create the contents of the kind of notes they wish to create. In contrast to the historic paper-based documentation workflow, the EHR user must painfully search through the bins of items buried in the software to extract the correct “pieces” of information necessary to complete the entry, requiring click after click after click in that process (Figure 1). While the freedom involved in creating paper notes might represent a positive, nostalgic memory, the healthcare system is not going to abandon EHRs with all the manifold advantages that they represent and provide.

In the Lego system, the myriad individual pieces (or modules) are assembled together by the rules (or protocols) dictated by the snap connections to create the toy version of an engineered system [4]. In creating a note, the user identifies and captures the necessary data pieces, analyzes and reassembles the pieces to assess the clinical situation at the level of complexity required, and develops a plan of action, thereby recreating a kind of clinical data system in itself each time a note is completed and entered [5]. But instead of rummaging around in a variety of bins for the right pieces, how could the de-binning ordeal be circumvented, and even improved, by a technical solution? We propose that a carefully engineered implementation of AI into the note creation software elements of the EHR would not only reduce the required rummaging through bins of pieces, but could assist in the assembly of those pieces into the desired output (ie, a useful, readable, and cogent note that meets all the necessary requirements for clinical documentation).

### Analyzing and Assembling the Pieces

In the context of EHRs, how can the natural, direct brain-to-hand workflow of paper note creation process be digitally recreated to simulate the free and seamless flow of information that historically emanates from the clinician’s brain directly onto paper? How could the obstructive middleman of technology be enhanced to support, rather than clog the process of clinical documentation? And could this be done in a fashion that makes utilization as intuitive as current Web browsers are to use? Furthermore, in addition to supplying the pieces, can this support also be applied to the assembly of the assessment and plan to assist in the production of a note that preserves the personal character—or the signature or “human”-ess—of the note writer? Optimally, in contrast to today’s copied and pasted rote entries, the production of a note that is more interesting and easier to read than current electronic notes would also be a goal of this redesign process.

We will progressively need to introduce important note information from other sources (eg, personal device and patient-entered data, population databases, even genomics) that supplement what is now available to the clinicians creating or reviewing the note at a later time [6]. In a previous publication, we described an engineered system that would support electronic note writing but did not specifically suggest how this might be done in a technical sense [7]. We suggest that the now increasingly familiar tools provided by AI provide a potential means by which to “de-bin” the process of data element selection and assist in the assembly of the data pieces with the goal of improved and more efficient electronic note creation.

AI has the potential to assist users in extracting the right information from the different information systems (ie, previous electronic notes and bedside monitors, and imaging, laboratory and pharmacy systems), assembling this information into the proper places in the note to assist in the formulation of the assessment with some bounds of certainty, and to analyze that assessment to develop a data-driven plan of action. There are many tools in the AI armamentarium—machine learning, natural language processing, computer vision, constraint satisfaction—but in essence, AI would power a learning interface between the human user and digital health information system to produce a note that would be highly, and increasingly over use, similar to that note-writer’s mental representation of what a clinical note should be.

We do realize that AI cannot analyze and repackage data until the latter has been incorporated into the system. The current history and physical examination, whether taken at the bedside or the office examining room, cannot be leveraged for note writing until they are so entered. Better, easier means for this must be devised: this might involve free text entry by voice recognition or keyboard, natural language processing of free text to enter structured data into the system, or new AI modalities as this exploding field develops.

Based on that current user input, as well as all available automatic data sources (eg, prior electronic notes, interfaced data like labs, and vital signs), AI would provide helpful suggestions to the user about what information is available and how it might influence the next course of action. AI could also function to emphasize or deemphasize certain elements of the record, based on previous results, external databases, and knowledge networks [8]. The technical strategy for providing these services could rest on a number of already available software solutions such as the tentative, but often very informative, textual suggestions that Google makes during searches. The careful use of autofill, especially for information types that tend to be repetitive, would cut down on excessive clicking and typing. Seamless but secure Internet connections integrated with the user interface would also facilitate decision support by allowing users to actively seek information not intrinsically provided even in these “smarter” applications. In addition, AI would help format and populate the note based on what has been “learned” from a user’s (and/or the patient’s) prior entries. There are tradeoffs and risks to innovation, but the quality of AI will intrinsically improve as it is employed in this regard, and the physician user will be the ultimate filter of what is saved and incorporated into the EHR.

### Creating an AI-enhanced Learning Healthcare System

At the heart of note writing is communicating important clinical events and decisions between different providers, and with the advent of patient portals, between providers and patients. AI is not the panacea to every problem in healthcare, but for a relatively repetitive and clearly defined task such as clinical note creation, it seems to provide a fairly ideal solution. It also bestows an opportunity to support an interdisciplinary care environment by learning from inter-specialty communication specifics and facilitating shared decision making by mining patient input and feedback. The final note would be the product of the user, but a user who is not exhausted by painful de-binning and endless clicking to insert the right data in the right places. An AI-enhanced system would boost the clinical workflow element of documentation, and maybe even inject some fun into the process of note writing. Such technology is upon us: 1 in 10 communications to the AI-powered personal assistant Amy (or Andrew) Ingram is a note of thanks, a testament to the 21st century computer passing the Turing test [9]. We certainly hope that an EHR company or some budding entrepreneur will take notice of this article and consider our idea in creating the next generation of EHRs.

## Abbreviations

AI: artificial intelligence
EHR: electronic health record
